# Machine Learning and Syncope Management in the ED: The Future Is Coming

**DOI:** 10.3390/medicina57040351

**Published:** 2021-04-06

**Authors:** Franca Dipaola, Dana Shiffer, Mauro Gatti, Roberto Menè, Monica Solbiati, Raffaello Furlan

**Affiliations:** 1 Department of Biomedical Sciences, Humanitas University, Pieve Emanuele, 20090 Milan, Italy; dana.shiffer@humanitas.it (D.S.); raffaello.furlan@hunimed.eu (R.F.); 2 Internal Medicine, Humanitas Clinical and Research Center—IRCCS, Rozzano, 20089 Milan, Italy; 3 IBM, Active Intelligence Center, 40121 Bologna, Italy; mauro_gatti@it.ibm.com; 4 Department of Medicine and Surgery, University of Milano-Bicocca, 20126 Milan, Italy; meneroberto@gmail.com; 5 Fondazione IRCCS Ca’ Granda Ospedale Maggiore Policlinico, 20122 Milan, Italy; monica.solbiati@gmail.com; 6 Dipartimento di Scienze Cliniche e di Comunità, Università degli Studi di Milano, 20122 Milan, Italy

**Keywords:** syncope, emergency department, diagnosis, risk stratification, artificial intelligence

## Abstract

In recent years, machine learning (ML) has been promisingly applied in many fields of clinical medicine, both for diagnosis and prognosis prediction. Aims of this narrative review were to summarize the basic concepts of ML applied to clinical medicine and explore its main applications in the emergency department (ED) setting, with a particular focus on syncope management. Through an extensive literature search in PubMed and Embase, we found increasing evidence suggesting that the use of ML algorithms can improve ED triage, diagnosis, and risk stratification of many diseases. However, the lacks of external validation and reliable diagnostic standards currently limit their implementation in clinical practice. Syncope represents a challenging problem for the emergency physician both because its diagnosis is not supported by specific tests and the available prognostic tools proved to be inefficient. ML algorithms have the potential to overcome these limitations and, in the future, they could support the clinician in managing syncope patients more efficiently. However, at present only few studies have addressed this issue, albeit with encouraging results.

## 1. Introduction

Artificial intelligence (AI) is a broad concept describing computer systems that can perform tasks considered to require ‘human intelligence’. Machine learning (ML) refers to the process of developing systems with the ability to learn from and make predictions using data without being explicitly programmed [1,2].

In recent years, ML has been adopted for solving complex problems in most sciences. In particular, it has also been promisingly applied in many fields of clinical medicine, such as radiology [3,4], dermatology [5], ophthalmology [6], and oncology [7,8].

In this narrative review, we summarize the basic concepts of ML applied to clinical medicine (Section 2) and explore its main applications in the emergency department (ED) setting (Section 3), with a particular focus on syncope management (Section 4).

Our objective was firstly to make a non-expert reader familiar with the elemental terminology and principles of ML. Secondly, through an extensive literature search in PubMed and Embase without language and time restrictions, we aimed to retrieve evidence on the use of ML algorithms in the field of emergency medicine, both for the diseases detection and prediction and for the patients risk stratification, triage, and disposition. Finally, for the first time, we analyzed the state of the art of ML possible applications in ED syncope management. For each topic, which we addressed in the corresponding subsection, we summarized the results of the studies we considered most significant, thus highlighting their main remarks and limitations. In the conclusions (Section 5) we reported the most relevant take home messages from the present review and possible future research directions.

## 2. What the Clinician Needs to Know about Machine Learning

Unlike most computer-based algorithms in medicine, which are ‘expert systems’ applying a set of known rules on a given topic to solve specific clinical questions, ML algorithms learn the rules from data [9]. Indeed, using clinical data from patients, ML algorithms can analyze a vast amount of variables, looking for combinations that reliably predict outcomes of interest. Traditional logistic regression techniques are commonly used to derive clinical decision rules (CDRs). Compared to those methodologies, the advantage offered by modern ML methods is the ability to use greater numbers of mathematical operations to better define complex relationships between risk factors and outcomes [1,2,9]. For example, in deep learning (DL), these operations are performed in layers; early layers perform mathematical operations to extract simple features, subsequent layers build on the simple features to generate more complex ones, and the final layer uses these features to make predictions [10,11].

ML is traditionally sub-classified as either supervised or unsupervised learning [1,2]. In ‘supervised learning’, labels are applied to the data and used for model development to determine a relationship between the input data and the label associated with the data. Examples include the automated interpretation of the ECG [12] or the detection of a pulmonary nodule on a chest CT-scan [13,14]. In this case, the machine is approximating what a trained physician could do with high accuracy. However, in modeling risk, the machine could find novel relationships not readily apparent to humans. In ‘unsupervised learning’, data are not explicitly labeled but, conversely, are classified by naturally occurring patterns or clusters. Although currently experimental, possible applications of these models deal with ‘precision medicine’, in which efforts are directed to redefine common and multifactorial diseases, according to their pathophysiological mechanisms, to provide new paths to therapy [15,16].

Supervised learning algorithms are typically used to predict a numeric value (regression) or a categorical value (classification). In linear regression, the assumption is made is that the predictor variables $X_{i}$ and the outcome variable Y can be related by a linear equation such as

$$Y=\beta_{0}X_{0}+\cdots +\beta_{n}X_{n}+\epsilon$$

The equation defines the desired model, and the algorithm objective is to identify through a process called learning (or fitting) the ‘optimal’ value of parameters $\beta_{i}$. If the relation is expected to be non-linear, more complex functions are used, like piecewise polynomials and splines. Particularly important is the case of logistic regression, one of the simplest models used for classification [17,18]. Notably, an important key objective is to keep the model as simple as possible (Occam’s razor). In this context this means not only to avoid unnecessary non-linearities but also to use only the predictors that are really required. Since comparisons between all possible predictors are typically computationally unfeasible and may lead to poor model generalizability, more advanced techniques like ridge regression and Lasso are used to reduce the number of parameters [19,20].

A crucial advantage of models like linear regression is that they are highly interpretable. If a coefficient $\beta_{i}$ is much higher than the other coefficients, we can consider the related predictor $X_{i}$ more important than the other predictors. A set of machine learning algorithms that share this key feature of linear regression is the set of tree algorithms, of which decision trees (DTs) are the basic block. Random forest (RF), an extension of DTs, is one of the most often used algorithms, at least for tabular data [21,22].

Support vector machines (SVMs) are a family of ML algorithms frequently employed because of their excellent performance [23]. Unlike regression and trees, they are more difficult to interpret and are based on the idea of separating data by means of hyperplanes or in a non-linear context by using more complex surfaces.

In the last decade, huge progress has been obtained by means of artificial neural networks (ANN) [24,25]. Such models are extremely powerful because they can be used to approximate a broad class of non-linear functions [26].

DL, also known as a deep neural network or deep neural learning, is a function of AI that mimics the workings of the human neural system to create patterns and process data for usage in decision making. As a subset of ML, DL has networks capable of learning unsupervised from data that is unstructured or unlabeled. DL models can perform classification tasks directly from images, text, or sound using neural networks and is therefore an extension of ANN. The peculiarity of these models consists in their multilayer architecture capable of learning the representation of data with multiple levels of abstraction. Indeed, each layer is obtained by composing non-linear functions that each transform the representation at one level, starting with the raw input (e.g., image array of pixels values), into a representation at a higher, slightly more abstract level (e.g., parts of familiar objects that combine into the final image). To date, DL proved to be extremely successful in handling images, notably with convolutional neural networks (CNN), and data sequences, notably with recurrent neural networks (RNN). Since they require very little engineering by hand and can benefit from increasing availability of large amounts of data, it is likely that, in the near future, such DL algorithms will be implemented with even greater success, despite limitations still existing regarding their explainability, interpretability, and traceability [10].

The choice of what ML model has to be used mainly depends on the type and amount of data available. DL methods (e.g., CNN and RNN) are generally used in assessing complex data like medical images or texts and large data sets. On the other hand, simpler ML systems (e.g., logistic regression or SVMs) require experts to predefine known discriminative features and actively help the algorithm to extract them [1]. However, a key trend in ML research is towards the automatic selection of the algorithm [27].

**Figure 1:**
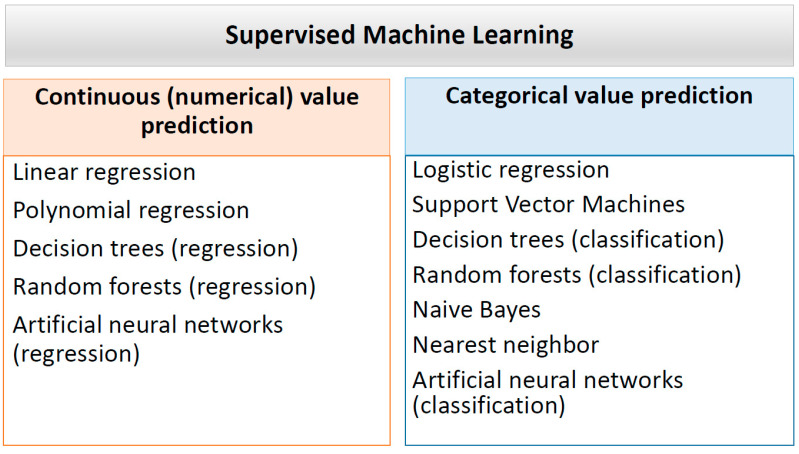
Overview of main supervised learning models. Please notice that the figure refers, for simplification, to the main applications of the different models but that all can predict continuous or categorical values depending on the variables of interest.

Main supervised learning methods are summarized in Figure 1.

The data set used to develop the ML model is called the development set. In general, it is split into a training set, in which the model’s internal values (i.e., parameters) are iteratively adjusted until the model optimally fits the data, and into a tuning set in which a set of predefined parameters (i.e., hyperparameters), are repeatedly adjusted, each time training a new model on the training set and evaluating that same model on the tuning set. After such ‘validation’, the model will have to be tested on a data set completely independent from the training and tuning set (i.e., validation set), to determine its generalizability and applicability in clinical practice [1]. It must be pointed out that some authors prefer using validation set rather than tuning set, and test set rather than validation set [28]. This is unfortunately, quite confusing.

It has to be pointed out that if an ML model is trained to predict the data too well, it cannot be generalized to other data sets. This overfitting can occur if large numbers of parameters are entered into the model, but they are not logically or biologically related to the outcome that the model aims to predict. Many data regularization techniques, such as data augmentation and early stopping, can be used to reduce overfitting [1].

In general, there are five main potential applications of ML algorithms to support clinicians and researchers:

- Detection: retrospective identification of patients with the disease from historical data (e.g., time series of medical device data).
- Diagnosis: identification of the disease from available information (notably, signs, symptoms, and tests results).
- Prediction: prediction of the future occurrence of a disease based on current and historical data.
- Prognosis: prediction of the future evolution of the disease based on current and historical data.
- Therapy: identification of the most appropriate therapy for the specific disease and patient; this is tightly related with the related need for personalization, particularly in the context of multi-morbidity.

An important distinction between detection and diagnosis is that for detection, it is a good practice to use any available information, while for diagnosis, only information that is expected to be available to the physician performing the diagnosis should be taken into account. Moreover, while for detection algorithms the lack of robustness and explainability may be acceptable, for diagnosis, it is of paramount importance to provide a comprehensive explanation of the algorithm ‘reasoning’ to the physician who takes the ultimate responsibility to decide whether to accept or ignore the ML recommendations. In addition, diagnostic algorithms must be robust to not change indications because of small, clinically irrelevant variations in the input data.

With the increasing use of electronic health records (EHRs), as well as digital imaging, there is currently a large amount of data that can be fed into ML models to improve diagnostic processes and patient risk stratification. Indeed, the automated analysis of natural language has been recently used to classify and extract clinically relevant information from patients’ clinical charts [29,30,31,32].

Natural language processing (NLP) is defined as computers’ ability to process spoken or written (natural) human language rather than mathematical equations or programs.

While quantitative data (i.e., vital signs and laboratory results) can be easily analyzed by computational systems, there is ongoing research on the use of free-text notes (i.e., clinical visit notes and reports of test results) with NLP and ML [30]. The Unified Medical Language System (UMLS), maintained by the National Library of Medicine, is a set of files and software that brings together health and biomedical vocabularies and standards to enable interoperability between computer systems [33]. These tools drove significant innovation in NLP use in medicine, implementing tasks such as disease classification in a clinical note or a medical textbook. Similarly, the Unstructured Information Management Architecture (UIMA) was developed by IBM for the analysis and search of unstructured data. The clinical Text Analysis and Knowledge Extraction System at the Mayo Clinic, built on the UIMA, was used to effectively extract information from the EHRs [34]. Liang et al. [31] developed an NLP-based diagnosis support system that enabled the identification of the most common pediatric diseases from EHRs. Their algorithm, tested on a large pediatric population (i.e., over 1,300,000 outpatient visits), was able to classify patients into pre-specified diagnostic categories with an accuracy comparable to junior physicians. Taggart et al. [32] compared two NLP methods for identifying bleeding among critically ill patients through the analysis of EHRs. They found that the rule-based NLP approach showed excellent performances in identifying bleeding, with high sensitivity and negative predictive value.

Considering all these premises, a near-future is conceivable in which ML algorithms will be able to increase doctors’ accuracy in diagnosing a disease or predicting its prognosis [9]. However, the lack of reliable diagnostic standards for many conditions, the need to preprocess unstructured high-value EHR data, and the need to develop and validate specific models for each pathology represent the main obstacles to this transformation.

## 3. How Machine Learning Might Help the Emergency Physician

The prompt interpretation of clinical data to classify patients and predict their outcome is crucial for the emergency physician. ED overcrowding decreases the efficiency of medical staff with a direct impact on cost and quality of care.

**Figure 2:**
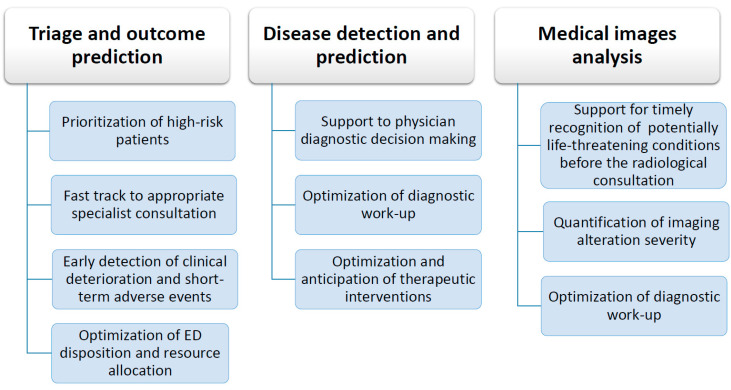
ML applications to ED operations and expected improvements. ED: emergency department.

ML techniques have the potential to improve ED operations in three areas (see Figure 2):

- Triage and outcomes prediction (prognosis support systems)
- Disease detection and prediction (diagnosis support systems)
- Medical images analysis

### 3.1. Triage and Outcomes Prediction

Several recently published articles highlighted how ML models could potentially improve triage operations in ED.

A RF model applied to triage data (vital signs, chief complaint, and active medical history) demonstrated equivalent or improved identification of clinical patient outcomes (need for critical care, an emergency procedure, and in-patient hospitalization) compared with traditional US triage methods based on the Emergency Severity Index (ESI) [35]. Recently, researchers at Massachusetts General Hospital developed four ML models (Lasso regression, RF, gradient boosted DT, and deep neural network) that all outperformed the ESI approach, both in critical care and hospitalization outcomes prediction [36]. However, the main limitation of these studies was the lack of external validation. Kwon et al. conducted a retrospective observational cohort study using nearly 11 million visits from the Korean National Emergency Department Information System. They found that a DL-based Triage and Acuity Score predicted in-hospital mortality, critical care, and hospitalization more accurately than traditional triage methods [37]. Unlike the other studies cited above, these authors included an external validation cohort; however, it is unclear whether their results can be generalized to Western countries. A recent systematic review [38], including 25 studies and 81 models, concluded that ML methods appear accurate in triaging undifferentiated patients entering the emergency care system. There was no clear benefit of using one technique over another; moreover, the majority of models’ reporting did not give enough information on development, validation, and performance, which makes a critical appraisal difficult.

Early prediction of hospital admission can optimize resources and allocation of beds as well as shorten the boarding times, thus limiting overcrowding. A Yale University research team [39] tested several ML models to predict ED disposition using clinical information from previous ED visits in addition to information collected at triage. They showed that ML could robustly predict hospital admission at ED triage, and the addition of patient history significantly improves predictive performance compared to using triage information alone.

ML models can also help emergency physicians to predict serious outcomes (i.e., mortality) and clinical deterioration. Analyses and predictions performed in ED are often limited CDRs, which use simple heuristics and scoring systems and suffer from poor generalizability. Taylor et al. [40] showed that an ML approach outperformed existing CDRs as well as traditional analytic techniques for predicting in-hospital mortality of ED patients with sepsis. In addition, ML classifiers significantly outperform clinical scores (qSOFA and MEWS) in screening septic shock among patients triaged for a suspected infection [41]. ML models proved to be also effective for early detection of patients at risk of cardiac arrest in ED [42,43].

*Clinical bottom line*—The integration of effective triage and patient outcome prediction could optimize ED operations by better matching the available resources to patients’ needs [44]. ML algorithms seem to outperform the traditional methods both in triage and in the prediction of ED patients disposition and outcome. However, their external validity and generalizability has yet to be confirmed before they can be used routinely.

### 3.2. Disease Detection and Prediction

A huge amount of data on patient demographics, symptoms, and clinical presentations of diseases are readily available in ED, as they are routinely generated, through the use of EHRs. This information can feed ML algorithms to support diagnostic decision-making in many disorders and promote faster and more effective therapeutic interventions.

Sepsis is one of the most challenging and resource-consuming conditions to diagnose and treat. Mao et al. [45] validated an ML algorithm with gradient tree boosting, InSight, providing high sensitivity and specificity for the detection and prediction of sepsis, severe sepsis, and septic shock using the analysis of only six common vital signs taken from EHRs (i.e., systolic blood pressure, diastolic blood pressure, heart rate, respiratory rate, peripheral capillary oxygen saturation and temperature). Similar results were confirmed by other authors [46,47]. Other areas in which ML models were successfully applied to diagnostic decision making include influenza [48,49], urinary tract infections [50], chronic obstructive pulmonary disease and asthma exacerbations [51], myocardial infarction [52], appendicitis [53,54].

*Clinical bottom line*—The possibility of automatically extracting clinical data from EHRs and comparing them with large clinical-administrative databases, constitutes an interesting perspective for the development of efficient ML based-diagnostic support systems. Despite the growing interest in research in this area, it must be pointed out that the lack of external validation constitutes the main limitation to the implementation of these diagnostic tools in clinical practice.

### 3.3. Medical Image Analysis

Presently, one of the most studied applications of ML models in medicine is emergency radiology. Emergency physicians are often tasked to recognize potentially life-threatening conditions from emergency scans before a radiologist’s review. In this setting, several ML models were developed and tested for head CT scan detection of hemorrhage, mass effect, hydrocephalus, and suspected acute cerebral infarction [55,56]. These showed high sensitivity and negative predictive value. Furthermore, DL algorithms proved to be effective in the recognition of traumatic bone fractures with human-level performances [57,58]. The recent Coronavirus disease 2019 (COVID-19) pandemic, which caused millions of deaths worldwide, led to ML algorithms’ application to detect and quantify pulmonary involvement in patients with pneumonia [59,60,61,62]. A DL algorithm for automatic detection of abnormalities in chest CT images from COVID-19 patients [62] showed higher sensitivity in comparison to radiology residents’ assessments and improved diagnosis efficiency by shortening the processing time.

*Clinical bottom line*—Radiology, thanks to the availability of a large amount of digitized images that can feed and train the algorithms themselves, currently represents the main field of application and research of ML and, specifically, of DL. In the emergency setting, the clear role in patient management and the presence of naturally occurring integration points within ED workflow will facilitate its diffusion in the next few years, despite the costs related to implementation and the technological disparity existing at the level of different hospitals.

## 4. How Machine Learning Might Help the Physician in ED Syncope Management

Syncope, defined as a transient loss of consciousness due to temporary global cerebral hypoperfusion [63], is a common symptom encountered in clinical practice and may manifest itself in a broad spectrum of conditions ranging from benign (i.e., vasovagal syncope) to life-threatening disorders (i.e., sustained arrhythmias, acute myocardial infarction, pulmonary embolism, aortic dissection). Syncope-related 7- to 10-day mortality risk is slightly lower than 1% [64], while the incidence of major adverse events at 30 days ranges from 5 to 17% [64,65].

Syncope is estimated to account for 1–3% of all ED visits and 6% of all hospital admissions [63]. A proportion of patients, ranging from 12% to 86% in different countries, are admitted to the hospital because of diagnostic uncertainty in ED, without a significant increase in the diagnostic yield despite a high economic resource expenditure [66,67]. An analysis by the US National Hospital Ambulatory Medical Care Survey from 2001 through 2010 documented over 3500 actual ED visits related to syncope, corresponding to approximately 1% of all ED visits. Admission rate for syncope patients ranged from 27% to 35% and showed no significant downward trend over time. The rate of non-diagnostic admissions remained persistently high across the 10-year study period, with over one-third of hospitalized patients discharged with the same diagnosis made on admission [68].

According to current international guidelines on Syncope management [63,69], the decision for hospitalization is primarily driven by the severity of the underlying disease or the presence of high-risk features identified during the initial evaluation in ED. On the other hand, inappropriate admissions in low-risk patients might increase risks related to hospitalization (including hospital-acquired infections and medication-related errors), and costs [70]. For patients deemed to be at intermediate risk, Syncope Unit (SU) management was recently proposed as an alternative to hospitalization [71]. The unit may be located in the inpatient or outpatient setting, with referrals coming from the ED or community practitioners/cardiologists. Two randomized clinical trials evaluated ED-based SU compared with usual care (i.e., hospitalization) [72,73]. They demonstrated higher diagnostic yield, lower hospital admission, reduced costs, and no increase in patients’ adverse outcomes randomized to the SU.

Therefore, if the etiology of syncope cannot be determined during the initial ED evaluation, an accurate risk stratification is crucial to ensure an appropriate ED disposition and optimize patients’ diagnostic–therapeutic pathway.

### 4.1. Syncope Risk Stratification and ED Disposition

Over the past 18 years, several syncope prediction tools were developed to guide clinician’s decision-making in the ED [74,75,76,77,78]. However, none of them proved superior to clinical judgment [79,80]. More recently, the Canadian Syncope Risk Score was developed and externally validated in a large population of Canadian patients, showing good discrimination and calibration for 30-day risk of serious adverse events after disposition from the ED. However, it needs to be validated in different settings before recommending its implementation in clinical practice [81,82].

Prognostic tools for syncope can be inefficient for a variety of reasons. Firstly, modeling the risk of a symptom that may be the expression of numerous diseases rather than a well-defined clinical condition can be difficult. Secondly, the available clinical decision rules are based on mean values obtained in patient groups; whereas, in clinical practice, decisions are personalized [83]. Finally, since syncope adverse events are rare, large cohorts of patients are needed to make a robust inference using traditional statistical methods.

ML could help overcome these limitations. However, to the best of our knowledge only a few studies explored this possibility. Costantino et al. [84] tested ANNs in the risk stratification of patients evaluated in the ED for syncope. They found that ANNs’ predictive accuracy was comparable, if not superior, to that of the currently available prognostic tools. In addition [25], ANNs could predict syncope patients’ hospitalization with a sensitivity of 100% and a specificity of 79%, potentially increasing the appropriateness of medical treatment and, consequently, hospital efficiency. However, the used methodology was constrained by small data availability, and no external validation of the model was performed.

Correct prognostic categorization of syncope patients may be challenging due to the high number of possible risk determinants and the ED physician time constraints. Since referring patients to a SU may not be feasible in all ED settings, it might be useful to have a rule-based system that reliably applies clinical guidelines to available data to assess the patient’s risk. Moreover, since most of the complex prognostic information is only available in textual form, a specific challenge is to apply NLP to EHRs to extract the relevant phenotypes.

*Clinical bottom line*—As it was demonstrated that nursing triage in ED can be inaccurate in identifying high-risk syncope patients [85], it is conceivable that the implementation of prognostic algorithms might lead to the future improvement of this ED operation, as already seen in other clinical conditions [86,87,88]. However, at the present state of the art, this application of ML has not yet been extensively studied and it is to be considered only experimental. In the next future, these technologies might provide patient’s individualized risk stratification thus helping ED physicians in their decision making.

### 4.2. Syncope Detection and Prediction

The diagnosis of syncope is often a highly complex exclusion process which is not sustained by specific diagnostic tests. Although the patient’s history, physical exam, vital signs, and the 12-lead electrocardiogram (ECG) may lead to an etiological diagnosis in up to 50% of patients [89], a high percentage of them still remain undiagnosed even after an extensive work-up. Often the lack of witnesses and/or the occurrence of retrograde amnesia in the patient, especially if elderly, can make it difficult to recognize the actual transient loss of consciousness. Therefore, experts recommend considering any alleged fall or unexplained loss of consciousness as syncope until proven otherwise [63,90].

However, the main objective of the diagnostic process is the identification or exclusion of serious and rapidly evolving conditions (i.e., sustained arrhythmias, acute myocardial infarction, pulmonary embolism, aortic dissection), of which syncope is just an epiphenomenon [90]. As discussed in the previous section, since the cause for syncope can be difficult to determine in the ED, risk prediction of short-term cardiovascular events and death is an important part of ED physician decision-making. The European and American syncope guidelines [63,69] defined in detail the diagnostic criteria and clinical features suggestive for cardiac and non-cardiac etiology of syncope. In addition, they both identified risk factors (from the medical history, physical examination, ECG, or laboratory test) for short- and long-term adverse events.

All these data, in structured or free text form, are routinely generated during patient visits and can be easily extracted from EHRs through the use of NLP-based algorithms. In a previous study [91], which was conducted on over 30,000 EHRs of patients evaluated in our hospital ED, we described a syncope detection algorithm based on natural language analysis applied to the episode reported by triage operators, the patient’s history and the ED physician evaluation, the ED discharge diagnosis description and the relative ICD 9 code. Overall, our SVMs classifier-based model was able to identify syncope patients with a sensitivity of 92% and a precision of 47%. In practice, this may significantly reduce the time necessary for the manual analysis of the charts, with low costs and high reproducibility in the Italian language. Although constrained by some limitations, including primarily the lack of external validation and in a language other than Italian, our algorithm could represent a valid tool for automatically selecting large populations of patients with syncope from clinical administrative databases. In turn, this might provide a massive amount of data suitable for prompt analysis and possibly real-time ED prognostic stratification. Recently [92], a tool developed through the selection by an RF algorithm of patient symptoms, past medical history, and witnesses reports differentiated syncope from other common causes of transient loss of consciousness—namely epilepsy and psychogenic non-epileptic seizures—with high sensitivity and specificity. RF classifier performed better than regression-based methods possibly because of the ability of ML model to exploit nonlinear interactions between predictors. However, this tool’s usefulness in clinical practice requires future confirmation since its accuracy decreases significantly in the absence of witnesses’ data; additionally, there is no external validation.

*Clinical bottom line*—To the best of our knowledge, no attempt has been made so far to develop a diagnostic support system for syncope. Due to the complexity of the syncope diagnosis, such a tool could be extremely useful in an ED context to identify neither low nor high-risk patients that should be referred to the SU for further investigation.

### 4.3. Life Parameters and ECG Monitoring

Repeated measurement of life parameters and ECG monitoring are key parts of the syncope work-up in ED [63,69,90].

In this regard, novel ML algorithms were developed, in an experimental setting, for the early detection of central hypovolemia and circulatory collapse by using collection and feature extraction, in real time, of arterial waveforms [93]. Also, the use of ML-enabled ECG, implemented by the use of wearable devices, is spreading in the cardiology field and is likely to increase diagnostic accuracy and efficiency by providing fully automated, unbiased, and unambiguous ECG analysis [94,95,96].

*Clinical bottom line*—Similarly to digital imaging, ML algorithms have recently also been successfully applied to the analysis and interpretation of various biological signals. These innovative technologies could find application in emergency medicine in the near future; however, at the present state of the art, there are no reports of their use in this setting nor specifically in the management of the syncope patient.

**Figure 3:**
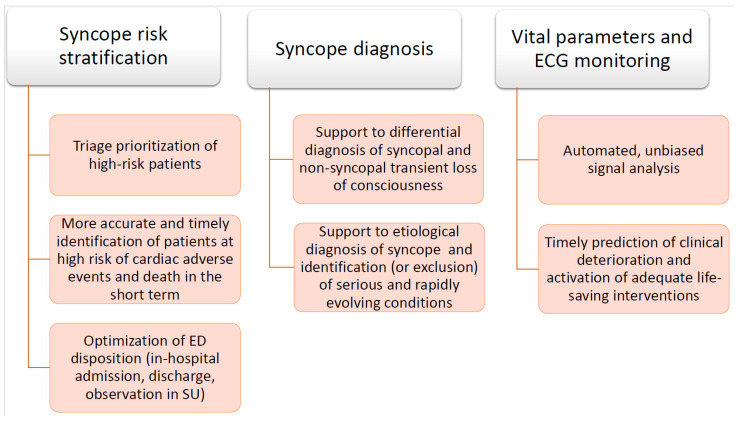
Potential applications of ML algorithms to the diagnostic work-up of syncope patients in the ED. ED: emergency department. SU: syncope unit.

Figure 3 summarizes the main future perspectives for the application of ML algorithms in the management of patients with syncope in ED.

## 5. Conclusions

In recent years, ML has been promisingly applied in many fields of clinical medicine, both for disease diagnosis and prognosis prediction.

Compared to traditional logistic regression techniques, ML algorithms can analyze a larger amount of clinical variables by performing greater numbers of mathematical operations and better define complex relationships between risk factors and outcomes of interest, in turn strengthening the predictive process. In addition, NPL algorithms allow to automatically extract data from patient EHRs, generated routinely during clinical activity, reducing the working time for the manual annotation of medical charts.

In the context of emergency medicine, extensive literature suggests how the use of ML algorithms can improve ED triage, diagnosis, and risk stratification of several diseases. However, the lacks of external validation and reliable diagnostic standards for many conditions reduce the quality of the evidence and currently limit these algorithms’ implementation in clinical practice.

Syncope is a frequent cause of evaluation in ED. It is often misdiagnosed, and none of the risk stratification tools currently available have been found to be more accurate than clinical judgment in predicting patients’ outcomes. Therefore, ML models in the future could support the emergency physician in managing these patients more efficiently. However, at present, only few studies have analyzed the application of ML to syncope detection and risk prediction, despite preliminary encouraging results.

Further and more robust evidence is needed before ML can actually support the emergency room physician in carrying out his daily clinical practice. Regarding syncope, research efforts will have to aim at the establishment and sharing of large clinical-administrative databases. The possibility to automatically extract and analyze risk factors from large patient populations could lead to optimization of patients’ diagnostic/therapeutic processes, ultimately improving the outcome, reducing inappropriate hospitalizations and overall health care costs. Finally, the implementation of these techniques, through data sharing, could generate significant progress in individual risk stratification and personalization of care.
